# Passing Messages between Biological Networks to Refine Predicted Interactions

**DOI:** 10.1371/journal.pone.0064832

**Published:** 2013-05-31

**Authors:** Kimberly Glass, Curtis Huttenhower, John Quackenbush, Guo-Cheng Yuan

**Affiliations:** 1 Department of Biostatistics and Computational Biology, Dana-Farber Cancer Institute, Boston, Massachusetts, United States of America; 2 Department of Biostatistics, Harvard School of Public Health, Boston, Massachusetts, United States of America; Niels Bohr Institute, Denmark

## Abstract

Regulatory network reconstruction is a fundamental problem in computational biology. There are significant limitations to such reconstruction using individual datasets, and increasingly people attempt to construct networks using multiple, independent datasets obtained from complementary sources, but methods for this integration are lacking. We developed PANDA (Passing Attributes between Networks for Data Assimilation), a message-passing model using multiple sources of information to predict regulatory relationships, and used it to integrate protein-protein interaction, gene expression, and sequence motif data to reconstruct genome-wide, condition-specific regulatory networks in yeast as a model. The resulting networks were not only more accurate than those produced using individual data sets and other existing methods, but they also captured information regarding specific biological mechanisms and pathways that were missed using other methodologies. PANDA is scalable to higher eukaryotes, applicable to specific tissue or cell type data and conceptually generalizable to include a variety of regulatory, interaction, expression, and other genome-scale data. An implementation of the PANDA algorithm is available at www.sourceforge.net/projects/panda-net.

## Introduction

Transcriptional regulation involves a number of distinct mechanisms that must work together to respond to internal or external stimuli [1]. Although the presence of transcription factor binding sites (TFBS) in the promoter or enhancer regions of a gene can suggest how that gene is controlled, not all TFBS are functionally relevant or active. Likewise, the binding of a single transcription factor (TF) alone may not be sufficient to recruit RNA polymerase, and several TFs may interact to promote or diminish regulatory potential. Epigenetic factors, post-translational modifications, stable and transient protein-protein interactions, and non-coding RNAs all likewise represent additional mechanisms that impact cellular regulatory networks. Considering how these various mechanisms might function together or independently to promote cellular activity is important when building comprehensive and interpretable network models.

The problem of gene network reconstruction has been well-studied and many computational methods to predict regulatory relationships and dynamics from a single data type exist (for example [2]–[5] also see [6] for a review of many existing methods). Reconstruction methods use various approaches including Bayesian network inference [7], [8] and ordinary differential equations [9]. Because of the large amount of gene expression data that is available, many methods attempt to use transcript levels to reverse engineer a regulatory network [10]. For example, one common approach is to use the mutual information among transcripts [2], [3]. Many methods examine the relationship between the expression levels of TFs and their potential targets to infer regulatory networks, either for individual targets or for larger regulatory “modules” 11–14. It has become clear, however, that network inference methods based on expression data alone are at best incomplete and often have trouble distinguishing between direct and indirect regulatory events [15]–[17]. Module-based network reconstruction methods can partially ameliorate this problem, but they tend to capture coarse-grained information corresponding to a few key regulators, recapturing large known regulatory pathways rather than new interactions [6], [18] and it remains difficult to obtain high-resolution regulatory information from gene expression data alone.

In contrast, integrative models incorporating multiple data types have been highly successful in other areas of bioinformatics [19]–[21] and they have begun to be applied to gene network reconstruction [22]–[25]. Many of these integrative models can incorporate data concerning promoter sequence information and protein-protein interactions [26] as well as mRNA expression levels [27] and ChIP-chip protein-DNA binding information (see, for example [28]). Integrative methods have been shown to perform better than those using any individual data type alone to accurately predict regulatory mechanisms [29]. As a consequence, a number of reconstruction algorithms include as inputs regulatory edges predicted *a priori* from external data sources such as sequence motifs [3], [30], [31]. Combining this sequence information or protein-DNA binding information from ChIP-chip/ChIP-seq experiments with epigenetic information regarding chromatin structure has become increasingly popular 32,33, especially in predicting networks for higher organisms such as mouse and human [34]. However, despite these significant advances in the field, it remains a challenge to effectively extract information from diverse data-types to recover genome-wide, condition-specific networks capturing accurate transcriptional regulator/target relationships [35], especially in higher eukaryotic organisms [36].

**Figure 1:**
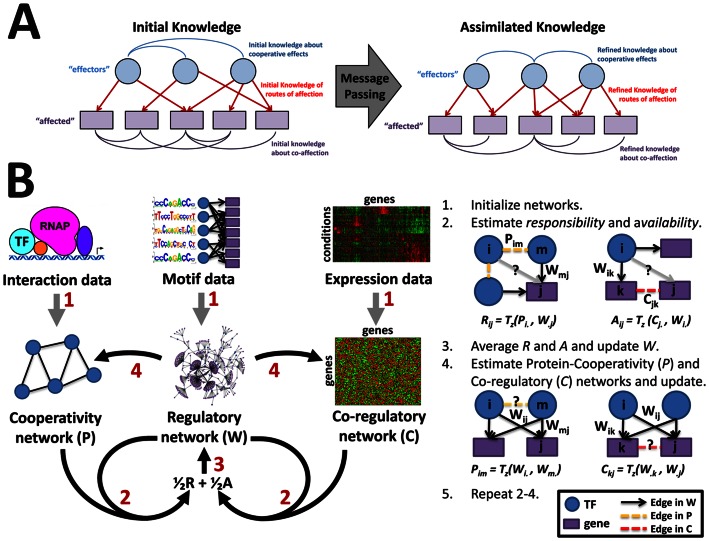
Outline of the PANDA approach for regulatory network inference integrating three data types. (**A**) A conceptual illustration showing the generalized framework for the message-passing procedure. (**B**) An illustration of how the message-passing procedure is applied in assimilating data that represents several various components of biological regulation. The networks are initialized from sequence motif data, physical protein interactions, and co-expression, respectively. The method iteratively passes messages within and among networks to emphasize agreement regarding the TF-gene regulatory relationships occurring within a system. At each time step regulatory (*W*), co-regulatory (*C*), and protein-cooperativity (*P*) networks are updated by passing information between the regulatory network, that reflects potential paths for regulation in the biological system, and the data-specific networks, that reflect “static” pair-wise information shared between gene products and TFs. At convergence, the method provides harmonized expression and interaction modules specific to a biological condition of interest, as well as the output regulatory network controlling those modules in each condition.

To overcome these limitations, we have developed a message-passing approach to systematically integrate information from different data-types. In the past, message-passing has been used to investigate combinatorial control in small networks using expression data alone [37], [38], to estimate signaling pathways by combining multiple sources of “omic” data [39], and to estimate the parameters in physical network models that incorporate protein-protein interaction, gene-expression and TF-gene interaction information [40]. In contrast to previous approaches, our primary goal is to pass information *between* multiple data-types in a meaningful and biologically informed way. To this end, we developed an algorithm, PANDA (Passing Attributes between Networks for Data Assimilation), that searches for *agreement* between different data-types by using the information from each to iteratively refine predictions in the others. This not only provides a more accurate gene regulatory network model, it also highlights the most informative aspects of the input biological data relevant to the network structure. A schematic view of the PANDA algorithm is shown in Figure 1 (see Methods for details).

We applied the PANDA algorithm to build condition-specific regulatory networks in *Saccharomyces cerevisiae*, or Baker’s yeast. We incorporated information regarding protein-protein interaction, gene expression and TF binding motif data and show that the resulting networks are not only more accurate, but also capture information regarding specific biological mechanisms and pathways that are missed using existing network inference methods.

## Methods

### Modeling Network Communication using Message Passing

The idea behind PANDA can be conceptualized by defining networks consisting of two types of nodes and three types of edges (Figure 1A). “Effector” nodes are agents that can in some way control the subsequent behavior of their “affected” targets. Edges can be drawn between either pairs of “effector” nodes, from an “effector” to an “affected” node, or between pairs of “affected” nodes. These three types of edges represent three sources of information that we consider in the model: 1) cooperative effects, or information about how the effectors may work together, 2) routes of affection, or simply which targets are affected by which effectors, and 3) co-affection, or information about how similarly targets are affected. In the past, message passing has been used to cluster data-points [41]. We instead use the message-passing procedure to assimilate the various initial information into one coherent model, passing attributes between the “effectors” and their “affected” targets along the various “routes of affection” and updating each until all three are in agreement with one another. This leads to refined information about cooperative effects, routes of affection, and how targets are co-affected.

The main objective of PANDA is to find *agreement* between the data represented by multiple networks. We will quantify this agreement with a heuristically defined similarity score based on the Tanimoto similarity [42] but with several minor modifications to better incorporate continuous z-score values as an input:
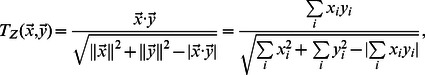
where 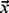 and 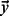 represent vectors of values normalized to z-score units. Given that 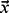 and 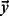 represent, as in our case, two sets of network edge weights, this will allow us to determine high similarity not only when we are confident that edges exist in both networks (two highly positive scores) but also when we are confident that edges do not exist in either network (two highly negative scores), allowing us to potentially fill in parts of the networks with sparse information. For more information regarding *T_Z_* as well as a discussion on a potential alternate form for this equation see the Methods S1 (also Figure S2D).

### PANDA: Passing Attributes between Networks for Data Assimilation

We apply the framework described above to estimate biological networks, where “effector” TFs can, through interactions with promoter regions, influence the behavior of “affected” genes. A schematic view of our approach is shown in Figure 1B. Rather than viewing information as flowing unidirectionally from TFs to their targets, we also imagine that additional elements associated with each target contribute to its ability to respond to the TFs that target it. Motivated by the concepts laid out by Frey and Dueck [41], we adopt the terminology of their method and for each edge define two quantities, the *responsibility* (*R_ij_*) which represents the information flowing from TF *i* to gene *j* and captures the accumulated evidence for how strongly the gene *j* is influenced by the activity of TF *i*, taking into account other potential regulators of gene *j*. Similarly, we define the *availability* (*A_ij_*) which represents information flowing from a gene *j* to a TF *i* and represents the accumulated evidence for how strongly the TF influences the expression level of that gene, taking into account the behavior of other genes potentially targeted by that TF. We note that the mathematical meaning of these terms presented here is different from the original Frey and Dueck paper.

We begin by creating a “seed” regulatory network (*W^(0)^*) to represent an initial estimate of the *total* availability and responsibility of the edges between TFs and their targets. This prior can be constructed using any source of regulatory information, including TF-gene regulatory interactions predicted by ChIP-chip or ChIP-seq experiments; however, due to the sparsity of such data, in the following analysis we choose to construct the network using motif information, creating an “edge” between TF *i* and gene *j* if the motif of TF *i* exists in the promoter region of gene *j*.

We also construct two other “seed” networks representing initial estimates of the probability that two genes are co-regulated by the same TFs and the probability that two TFs cooperate to regulate common genes. Specifically, we create a co-regulatory network (*C^(0)^*) defined by Pearson correlation coefficients between the expression profiles of gene pairs. Further, we recognize that the transcriptional regulatory mechanism involves multiple interacting factors that cooperate together to initiate the transcription of a gene. Although there are multiple ways proteins can cooperate to activate a gene, one primary mechanism is by the formation of a physical protein complex. Thus, we use physical protein-protein interaction data to define pairs of TFs that cooperatively regulate genes in *P^(0)^*. The three initial “seed” networks are normalized such that their edge weights are represented by Z-scores (see Methods S1). The following message-passing approach maintains this interpretation of edge weights.

We combine the regulatory network with the protein-cooperativity network to predict the *responsibility* (*R_ij_^(t)^*) of an edge from TF *i* to gene *j* in the regulatory network. Namely, since TFs that cooperate together share responsibility for regulating the same set of genes, at each iteration, *t*, we determine the level of agreement between the TFs that target gene *j,* (*W_.j_^(t)^*), and those that cooperate with TF *i,* (*P_i._^(t)^*):
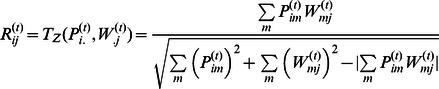



In the same manner we combine information in the regulatory network with the co-regulatory network to predict the *availability* (*A_ij_*) of an edge between TF *i* and gene *j* in the regulatory network. Namely, since genes that are targeted by the same TF are co-regulated, to calculate *A_ij_^(t)^* we determine the level of agreement between the regulatory targets of TF *i* (*W_j._^(t)^*) and the set of genes with which gene *j* is co-regulated (*C_.j_^(t)^*):
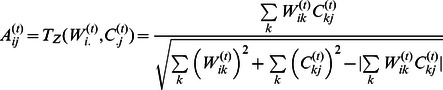



Since regulation requires both that a TF is responsible for the regulatory status of its target gene and that the target gene is available to be regulated by that TF, we use the average of these two values (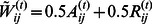) and update the regulatory network by a small amount (*α*; *0<α<1*):
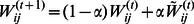



We pass messages not only between TFs and their targets but also among different data-types. Namely, since TFs that target the same sets of genes are likely to cooperate together when regulating those genes, we can estimate the weight of an edge between two TFs, *i* and *m*, in the protein-cooperativity network (*P_im_*) by comparing the set of genes regulated by TF *i* to those regulated by TF *m*:
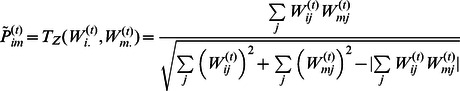



Similarly, since co-regulated genes are, by definition, targeted by the same TFs, we estimate the weight of an edge between two genes, *j* and *k*, in the co-regulatory network (*C_kj_*) by comparing the set of TFs targeting gene *k* (*W_.k_*) with the set of TFs targeting gene *j* (*W_.j_*):
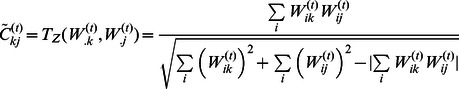



This process gives estimates for the protein-cooperativity and co-regulatory networks that are in agreement with what is known about the regulatory interactions (*W_._*) that we use to update *P_._* and *C_._*:
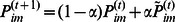


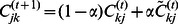



In the following analysis we set the update parameter, *α*, equal to 0.05. We note here that results of PANDA are consistent across a wide range of values for *α* (see Figure S1A). These updates (of the regulatory then cooperativity and co-regulatory network edge weights) are iteratively repeated. Over time self-co-regulation (*C_jj_*) and self-cooperativity (*P_ii_*) increase relative to other co-regulatory or cooperative events, guaranteeing convergence. For additional details regarding the PANDA algorithm and the motivation behind it see the Methods S1.

## Results

### PANDA Recovers Edges in Simulated Networks

We initially tested the PANDA algorithm on simulated data. To that end, we simulated 100 random networks to represent “true” routes-of-affection by generating 500 random connections between 25 “effector” nodes and 100 “affected” targets. For each of these randomly generated routes-of-affection networks, we determined the “true” accompanying cooperative-effects and co-affection networks by connecting effectors if they both targeted more than two of the same targets and “affected” targets if they share any of the same “effectors”, respectively. We then added noise to each set of these networks (cooperative effects, routes of affection and co-affection) by performing an edge randomization. For the routes of affection network we performed 125 “edge swaps” and for the cooperative effects and co-affection networks we performed a number of swaps equal to 50% of the number of edges in the networks. As a result, we obtained 100 sets of noisy initial networks to submit to PANDA, and 100 sets of the original “true” networks with which to evaluate PANDA’s performance.

We evaluated the performance of PANDA on our simulated data by determining the area under the receiver operating characteristic curve (AUC-ROC, hereafter shortened to AUC). We calculated the AUC values for the initial “noisy” networks submitted to PANDA as well as the AUC values for each of PANDA’s final predicted networks, using our original “true” networks as a gold standard. The median and standard deviation of these values across our 100 randomizations are reported in Table 1. We observe a clear increase in the AUC in all three networks. In order to evaluate the significance of this increase in AUC, for each network, we took the difference between the final and original AUC values across all the randomizations, fit the results to a normal distribution, calculated the standard-score, and report the associated *p*-value (see Methods S1 for more details). All three networks significantly improve. The most significant improvement was found in the co-affected network and the least significant improvement was found in the cooperative-effects network. We believe this may be partially attributable to the differences in network size as the cooperative-effects network only contains 25 nodes and 300 possible connections, whereas the cooperative-effects network contains 100 nodes and 4950 possible connections.

**Table 1:** PANDA is able to recover information lost via adding noise to simulated networks.

Network	Initial AUC (med.±σ)	Final AUC (med. ±σ)	Significance
**Cooperative Effects**	0.587±0.028	0.662±0.036	0.024
**Routes of Affection**	0.756±0.009	0.789±0.010	1.67e-4
**Co-affected** **Targets**	0.566±0.008	0.643±0.011	2.49e-11

Values represent the median and standard deviation across 100 randomizations in which an “effector” to “affected” target network was generated, and the “true” network representing cooperative effects and co-affected targets based on this network was calculated. Noise was added to each network and the noisy networks were submitted to PANDA. The AUC was calculated by comparing the final networks predicted by PANDA to the original “true” networks. Significance was determined by fitting the difference between the original and final AUC to a normal distribution.

### PANDA Improves upon Initial Estimates for Regulatory, Co-regulatory and Protein-cooperativity Networks in Yeast (*Saccharomyces cerevisiae)*

As an initial evaluation of our algorithm on biological data we collected expression data for TF knock-out or over-expression conditions in *Saccharomyces cerevisiae* [43], [44]. These data were combined with the known locations of TF motifs in sequence data [45], [46] as well as with a comprehensive set of Affinity Capture-MS protein-protein interactions from BioGRID [47], [48]. These data defined our initial co-regulatory, regulatory and cooperativity networks, respectively. The following analysis was run using information for 53 TFs and 2555 genes for which we had information across the different data-sets used (for more information, see Table 2 and Methods S1).

**Table 2:** Data used to construct both the initial and gold-standard networks used in the evaluation of PANDA and the other network reconstruction algorithms.

Network Name	Data used to construct network[reference]	Number of TFs/Genes/Conditions
Initial Cooperativity Network ( 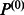 )	Affinity Capture-MS [47], [48]	TFs: 53 (43 with evidence)
Gold Standard Cooperativity Network ( 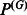 )	Low-throughput evidence [47], [48]	TFs: 33
Initial Regulatory Network ( 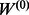 )	Motif [45], [46]	TFs: 53 Genes: 2555
Gold Standard Regulatory Network ( 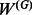 )	ChIP-chip [45], [49]	TFs: 52 Genes: 1073
Initial Co-regulatory Network – Knock-Out Data ( 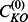 )	Gene Expression [43], [44]	Genes: 2555 Conditions: 106
Gold Standard Co-regulatory Network ( 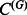 )	ChIP-chip [45], [49]	Genes:1073 (1072 co-targeted)
Initial Co-regulatory Network – Cell Cycle Data ( 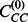 )	Gene Expression [51]–[54]	Genes: 2555 Conditions: 56
Initial Co-regulatory Network – Stress Response Data ( 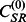 )	Gene Expression [55], [56]	Genes: 2555 Conditions:173

References include both the publication and the website from which the normalized data was downloaded. The number of transcription factors and genes reported are those used to construct each network. The 53 transcription factors and 2555 genes mentioned in the initial protein-cooperativity network, initial regulatory network and initial co-regulatory networks are the same set of TFs and genes for all networks, and represent those for which we had both motif and expression data. For the initial cooperativity network, we allowed TFs for which we had no Affinity Capture-MS data to be initialized as self-cooperating (*P_ii_ = 1*). The transcription factors and genes used to construct the three gold-standard networks are the subset of the aforementioned 53 TFs and 2555 genes for which we had ChIP information (52 TFs and 1073 genes), or “low-throughput” interaction information (33 TFs). We used this subset of TFs and genes when evaluating the quality of each network. “Low-throughput evidence” data represents interactions with evidence from “co-fractionation”, “co-localization”, “FRET” or “reconstituted complex.”

We ran PANDA using these three initial networks and observed convergence after approximately 120 iterations of message passing (see Figure S1B). Before examining the properties of the predicted protein-cooperativy or co-regulation networks, we first focused on the quality of the predicted regulatory network. We determined the AUC value using experimentally-defined TF binding sites identified using ChIP-chip [45], [49] (p<10^−3^) as a “gold standard.” Since the motif and ChIP data were published by the same lab, we attempted to ensure independence between the prior and validation set by using motif data that had not been filtered either by sequence conservation or from the results of the ChIP experiments [46]. In all subsequent evaluations the AUC is calculated using only the subset of edges between genes and/or TFs for which we had information in our “gold standard.”

**Figure 2:**
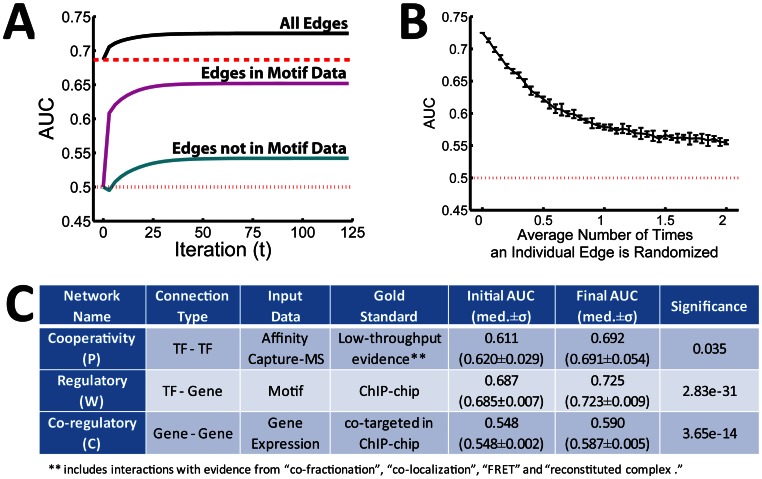
An evaluation of PANDA’s performance. (**A**) The significance of the area under the ROC curve (AUC) for the regulatory network predicted by PANDA at each step during convergence using all experimental data types. Red dotted and dashed lines indicate the AUC values for either random (0.5) or the motif prior (0.687), respectively. Edges included in the motif prior as well as those that are not included in the motif prior are evaluated separately. As messages are passed, the quality of the regulatory network increases. A large portion of this improvement is attributable to a removal of false positives from motif-edges. (**B**) PANDA’s performance as noise is added to the motif prior. Even upon “full” randomization of the initial motif network, PANDA is able to improve the network prediction, indicating that it can still find biological signal in the absence of an accurate prior. (**C**) Evaluation of the accuracy of transformation for each data type specific network by PANDA. The initial AUC of each input network is shown as well as the AUC of the edges predicted by PANDA. The significance was determined by jackknifing the input data.

We note that motif data alone is already moderately predictive of the regulatory network as determined by our ChIP-chip edges (AUC = 0.687). However, at each step PANDA is able to improve upon this initial estimate (Figure 2A), increasing the quality of our predicted regulatory network and resulting in a final predicted regulatory network of higher quality (AUC = 0.725) than motif data alone. To better understand whether a removal of false positives from edges that were in the prior motif network or the removal of false negatives from edges that were not in the prior motif network most strongly contributed to this increase in quality, we also separately tracked the AUC for the subset of edges belonging to the motif data and the remainder that does not. Figure 2A demonstrates that although both subsets of edges improve in quality after iteration, the majority of the overall AUC improvement is a result of the removal of false positives from the motif prior. This is heartening as a common practice in interrogating networks is to focus on a certain number of “top” edges – in which case removal of false positives from these edges can play a significant role in improving the network.

We also wished to test the sensitivity of PANDA’s performance to the quality of the various input data- types. First, we randomized the gene labels in the input expression data matrix. We calculated the mean and standard deviation of the final AUC over 100 such randomizations and found that even in the absence of informative expression data we are able to improve the quality of the network relative to the motif prior (final AUC = 0.712±0.001 compared to 0.687), indicating that the prediction of individual regulatory edges can be improved upon by considering only protein interactions and the local network structure. Since the motif data alone has a significant effect on the quality of the final network, we also tested how PANDA would perform with the addition of noise in this prior data. To add noise, we randomly “swapped” network edges a certain number of times, while keeping the degree of the genes and TFs fixed. We varied the number of “swaps” and report the mean and standard deviation of ten randomizations for each probability that an individual edge is “swapped” in the randomization (Figure 2B). As the prior is randomized, the AUC of the final predicted network, as expected, decreases. Upon “full” randomization of the motif prior, the AUC of the final network is approximately 0.55, illustrating that PANDA is able to predict informative networks even in the absence of accurate initial regulatory information.

Finally, to determine the significance level of the improvement in AUC, we used a jackknife procedure in which we removed motif, interaction and expression data regarding a random 10% of TFs and genes and ran PANDA on the remaining data. We repeated this 100 times, fit the results to a normal distribution (median and standard deviation of the AUC values across these jackknifed networks are reported in Figure 2C), calculated the standard-score, and reported the associated p-value for improvement (see Methods S1 for more details). For the regulatory network the improvement in AUC is very statistically significant (p = 2.8×10^−31^).

In addition to a regulatory network (*W_ij_*), PANDA also refines two other networks representing TF-cooperativity (*P_ij_*) and gene co-regulation (*C_ij_*). We hypothesized that PANDA could help identify the functionally important interactions in these co-regulatory and protein-cooperativity networks. With this in mind we selected a “standard” by which to evaluate the networks predicted by PANDA representing these two other data types. For the co-regulatory network we constructed a standard using the identified ChIP-chip interactions, assigning each gene-pair a value of 1 if both members of the pair have a binding site associated with a particular TF, and 0 otherwise. To create a “high-confidence” evaluation set for the protein-cooperativity network, we selected interactions within the BioGRID database [47], [48] that have been validated via stringent criteria, including “co-fractionation,” “co-localization,” “FRET,” or “reconstituted complex.” The AUC of the initial networks we submitted to our message-passing algorithm as well as the AUC of the final networks predicted by the message-passing algorithm, based on these standards, is shown in Figure 2C. Both the co-regulatory and protein-cooperativity networks grow closer to the chosen standards as the message-passing occurs. The protein-cooperativity network sees a significant increase in AUC, from 0.611 to 0.692 (p = 0.035). The co-regulatory network does not see much of an increase in AUC value (0.548 to 0.590); however, this improvement is still highly significant (3.7×10^−14^) given the size of the network that we are evaluating and the standard we are using.

### PANDA Learns more Accurate Regulatory Networks for Yeast than Existing Reconstruction Approaches

Next we compared the quality of the network predicted using PANDA with networks predicted using the same input data by four commonly used network reconstruction algorithms: SEREND [50], which employs a semi-supervised learning method, ReMoDiscovery [11], which uses a module reconstruction method, and CLR [2] and C3Net [4], both of which use mutual information in gene expression to predict a regulatory network. For a broader understanding of the types of biological networks each algorithm may be tuned to predict, we downloaded two additional expression datasets: a time-course experiment in which the expression levels of synchronized cells were measured through several cell cycles[51]–[54], and a collection of experiments in which gene expression levels were measured after exposing yeast to stress-inducing conditions including heat shock and starvation [55], [56](for more information, see Table 2 and Methods S1).

**Figure 3:**
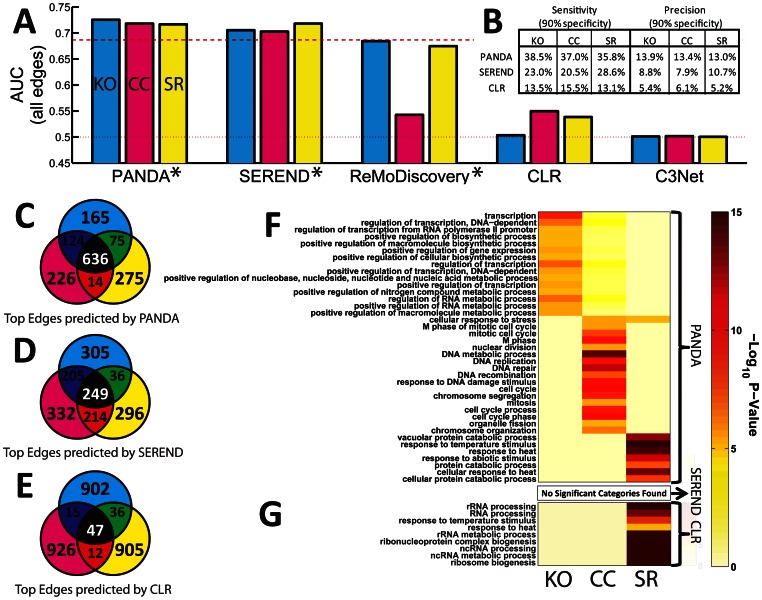
A quantitative and qualitative comparison of the networks predicted by PANDA to those predicted by other network reconstruction algorithms. (**A**) The AUC of the networks predicted by PANDA, SEREND [50], ReMoDiscovery [11], CLR [2] and C3Net [4] using three distinct sets of expression conditions as an input: regulator knock-out (cyan), cell-cycle (magenta) and stress-response (yellow). PANDA, SEREND and ReMoDiscovery all take prior regulatory information from motif data as an input (indicated with an asterisk) whereas CLR and C3Net derive networks using only expression data. Red dotted and dashed lines indicate the AUC values for either random (0.5) or the motif prior (0.687), respectively. (**B**) The sensitivity and precision at 90% specificity reported for the condition-specific networks predicted by PANDA, SEREND and CLR. By this measure, PANDA outperforms both SEREND and CLR. (**C–E**) The overlap of the top 1000 edges by weight from the networks predicted by (**C**) PANDA, (**D**) SEREND, or (**E**) CLR using three distinct sources of gene expression. Many edges are common between the networks predicted by PANDA, but there are also subnetworks of edges unique to each data type that may highlight distinct regulatory programs. In contrast to PANDA, fewer edges are common between the networks predicted by SEREND and almost none are in common between the networks predicted by CLR. (**F–G**) Functional analysis of genes belonging to each of the condition-specific subnetworks identified with (**F**) PANDA or (**G**) CLR. GO categories enriched at Benjamini-Hochberg FDR less than 10^−5^ and which contain at least 10% of the members in one of the condition-specific gene sets are shown. No categories were enriched at this level for genes belong to the condition-specific subnetworks identified by SEREND.

We ran SEREND, ReMoDicovery, CLR, and C3Net on these three expression data-sets (regulator knock-out, cell-cycle and stress-response) using their default parameters and compared with PANDA. The AUC for the networks produced by each algorithm is shown in Figure 3A. As before, AUC was calculated using only the subset of edges between TFs and genes for which we had information in the ChIP-chip “gold-standard.” Overall, PANDA performed well with a final overall AUC of approximately 0.72 in all three datasets. Both SEREND and ReMoDiscovery integrate both motif and expression data, so, not surprisingly, their overall performance is more similar to PANDA compared to CLR and C3Net. SEREND does slightly worse than PANDA with a final overall AUC of just over 0.70 in the knock-out and cell-cycle datasets, but performs similarly to PANDA in the stress-response data set (AUC of 0.718). ReMoDiscovery predicts networks with a fairly high overall AUC of about 0.68 using both the knock-out and stress-response sets of conditions; however, the algorithm begins with a motif prior that alone already has an AUC of about 0.69, so actually the addition of the expression data hurts the predictive power of the final network relative to the initial network.

CLR and C3Net performed relatively poorer, due largely to the fact that these two approaches only consider gene expression data. Despite this limitation, CLR is able to predict ChIP-chip edges with an AUC of about 0.55 (similar to PANDA’s performance using randomized motif data) in the cell-cycle and stress-response datasets, but it performed poorly using the set of knock-out expression conditions. C3Net is unable to estimate a predictive regulatory network using these three sets of expression conditions.

Since the inclusion of motif data has such a strong influence on the final AUC values, we de-coupled this information from the networks predicted by each, performing the AUC analysis twice more, evaluating the edges that exist in the initial motif network (edges for which *W_ij_^(0)^* = 1) and those that don’t (edges for which *W_ij_^(0)^* = 0) separately (Figure S2A–B). Within the edges for which there is motif information PANDA vastly outperforms the other algorithms, with an AUC of about 0.63–0.65 compared to 0.48–0.55 for SEREND, 0.48–0.5 for ReMoDiscovery, 0.53–0.55 for CLR and 0.5 for C3Net. Within the edges that are not supported by motif data PANDA performs slightly better than ReMoDiscovery, CLR and C3Net, with an AUC of around 0.53–0.55 compared to 0.5 for ReMoDiscovery, 0.46–0.53 for CLR and 0.5 for C3Net. Interestingly, SEREND does fairly well on non-motif edges, with an AUC of around 0.58–0.59. We note that we surprisingly find some AUC values below 0.5, representing an edge weight estimation which is *worse* than random. In these instances ordering the edges by their predicted weights does not create a random ordering, but one which is in the opposite direction as the gold-standard. Since this “reverse” ordering is generally found either for edges in the motif prior or for edges not in the motif prior, we hypothesize that sometimes an algorithm might be very effectively improving the classification of one type of edge, but does so at the expense of reversely classifying the opposite type.

These above results indicate that the overall superior performance of PANDA relative to the other reconstruction algorithms is not attributable to integration of motif data alone but that *how* PANDA integrates this data is also critical to the model’s performance. Namely, by considering the neighborhoods surrounding both “ends” of a regulatory event, i.e. both the cooperating partners of a TF and co-regulatory partners of a gene, PANDA is able to better estimate the potential for that regulatory event to occur.

PANDA, SEREND and CLR all showed improved performance relative to their initial network configurations, so in the following analysis we will investigate and compare the functional properties of these networks. However, first, since the AUC is only a coarse measure of performance, we calculated the sensitivity and precision of the networks predicted by each algorithm at a 90% specificity level (Figure 3B). By this measure, PANDA clearly performs better than both SEREND and CLR, with a sensitivity ranging from 36% to 39% and a precision of 13–14% compared to 23–29% sensitivity and 8–11% precision for SEREND and only 13–16% sensitivity and 5–6% precision for CLR. We also repeated this analysis on networks reconstructed using expression and motif data. We did this by running PANDA without input protein-protein interaction data ((*P_ij_*) is initialized to the identity matrix), SEREND as before, and integrating motif information with the final CLR predictions (see Methods S1). The results of the analysis are shown in Figure S2C. When the exact same data is used for all three reconstruction techniques, we still observe that PANDA outperforms both SEREND and CLR. PANDA performs at a level only slightly below the previous analysis (sensitivity around 35–38%, precision 13–13.7%). The addition of motif information enhances CLR’s performance to approximately the same level as SEREND (sensitivity 26–27.5%, precision around 10%), but both do not perform as well as PANDA, even in this context.

### PANDA Accurately Predicts Condition-specific Functional Information for Yeast

Cellular networks are known to alter their topology in response to external and internal conditions and stimuli. It is therefore vital that the networks predicted for these diverse systems are not only accurate, but also are representative of the specific biological pathways of the system in question. With this in mind we determined the functional properties associated with genes targeted in the condition-specific networks predicted by PANDA, SEREND and CLR (functional analysis using the CLR plus motif integrated network varies little from the results discussed below and is included in Figure S3).

Since none of the three conditions represented by our networks are lethal, we expect that those networks will share common pathways essential for cell viability. To test this hypothesis, we selected the 1000 top edges (between TFs and genes) by weight in each of the three condition-specific networks predicted by PANDA, SEREND and CLR (Figure 3C–E, see also Figure S2E). For the networks predicted by PANDA we find that many edges are common, reflecting common regulatory mechanisms. SEREND also has a considerable overlap in its top predicted interactions, although only about half as many as PANDA. In contrast, the top-weight edges predicted by CLR in each of the three networks are very divergent. These results indicate that PANDA may be identifying pathways essential to cell viability in addition to the particular ones highlighted in each set of expression conditions.

For the networks predicted by PANDA, SEREND and CLR, we identified subsets of edges that are specific to each condition and used them to define nine condition-specific subnetworks (three each for PANDA, SEREND and CLR). We then determined the set of genes represented in each of these nine subnetworks and used DAVID [57] to evaluate which Biological Process GO categories were enriched in these nine sets of genes. Figure 3F includes all GO categories enriched in any of the gene-sets derived from PANDA’s predicted networks, where enrichment is defined as having at least a 10% overlap in genes and a Benjamini-Hochberg false discovery rate (FDR) of less than 10^−5^. The genes contained in the subnetwork corresponding to the knock-out expression conditions are enriched in processes such as “transcription” and “positive regulation of gene expression,” consistent with general dysregulation caused by perturbation of TF activities. In contrast those genes selected based on their connectivity in the network predicted using time-series data from synchronized cells are associated with functions related to the cell cycle, and those genes selected based on their connectivity in the subnetwork specific to the stress-response dataset are associated with stress-related functions such as “response to heat” and “response to antibiotic stimulus.” “Cellular response to stress” is enriched both in genes associated with the time-series and stress-response datasets, which is not surprising since in both cases the cells are undergoing stressful conditions, either through forced synchronization or by exposure to harsh conditions.

In contrast, it is harder to discern any functional role for the genes in the condition-specific subnetworks predicted by SEREND or CLR (Figure 3G). No GO categories were enriched in any of the condition-specific gene sets derived from the SEREND subnetworks or for the sets of genes identified in the regulator knock-out and cell-cycle related networks predicted by CLR. The genes belonging to the CLR subnetwork of edges corresponding to the stress-response dataset are enriched in several GO categories, but most of the identified categories are nonspecific such “ribosome biogenesis” and “RNA processing”.

Since often a laboratory may not have the luxury of contrasting networks built using such heterogeneous expression conditions, we repeated the functional analysis using all top edges to construct our genes sets (Figure S3B). In this case, the overall significance of the identified categories decreased, but PANDA was still able to identify condition-specific pathways for both the cell cycle and stress response networks, while the same analysis still yielded no identified categories for SEREND and approximately the same categories for CLR (not surprising given the low edge overlap, see Figure 3E). This analysis also shows that PANDA can still find highly condition-specific information even when just one dataset is available. We also note that the results of the functional analysis are quite robust and we recover similar information even when using a lower threshold to select the functional categories (Figure S3C).

### PANDA Uncovers Condition-specific Regulatory Modules for Yeast

**Figure 4:**
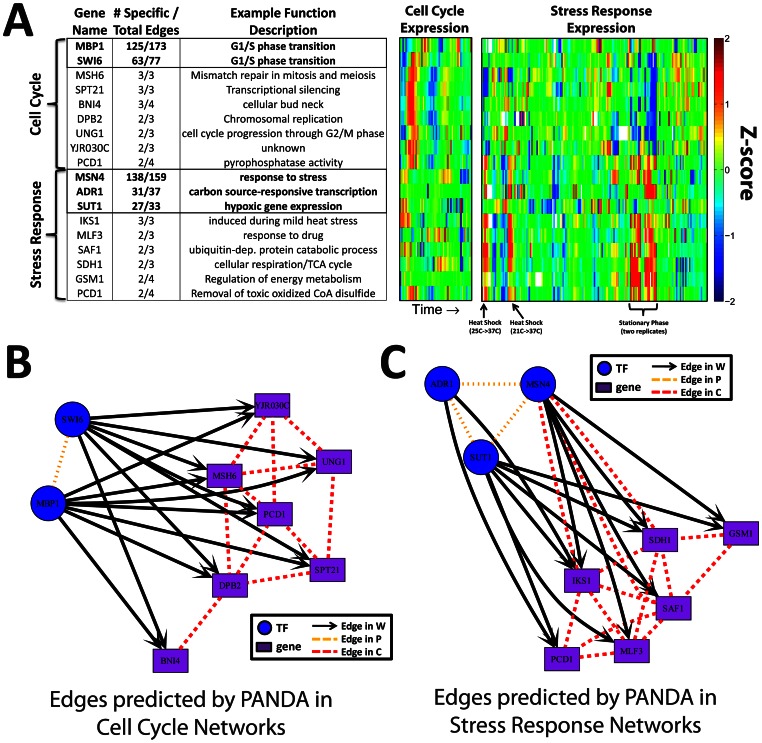
Specific examples of condition-specific genes and edges highlighted by PANDA. (**A**) A table of genes (transcription factors bolded) with an enrichment of edges in a particular condition-specific network compared the union of all the networks and an example of their functional role in the cell. Also, the expression levels of these genes across the conditions in the cell-cycle and stress-response datasets. For visualization purposes, each row in each dataset was normalized to a Z-score. The co-expression of the genes in these regulatory “modules” is easily discernible. As co-expression between genes and transcription factors is not used by PANDA when building the networks it is not surprising that some of the TFs are not as highly co-expressed with the other identified genes. (**B–C**) Visualization of the edges surrounding these enriched genes in the (**B**) cell cycle and (**C**) stress response condition-specific networks. Co-regulatory (*C*) and protein-cooperativity (*P*) network edges are shown if they are in the top 10% of edges identified by PANDA in the final condition-specific co-regulatory and protein-cooperativity networks (for more information see Methods S1).

To better capture gene-level regulatory information uncovered by PANDA, we identified genes and transcription factors for which a large proportion of their adjacent edges are uniquely identified in either the cell-cycle or stress-response networks predicted by PANDA. We also identified their associated regulatory events, and used this information to construct regulatory modules (see Methods S1). A sample function as well as the expression levels of the identified genes across the cell cycle time course and stress conditions is shown in Figure 4A (transcription factors noted in bold).

The functional and co-regulatory behavior of the selected genes demonstrate that using PANDA’s predicted networks can identify highly-specific condition-driven cellular events. For example, the genes associated with the cell cycle network, with the exception of MBP1, are clearly synchronized across the time-course and the genes highly targeted in the stress response network, with the exception of SUT1, are visually correlated across the various stress conditions. These stress-associated genes are highly expressed in the stationary phase, when yeast has depleted all available nutrients and consequently halts the cell cycle. The fact that the genes identified based on PANDA’s cell cycle network have lower expression in these conditions serves as an independent validation of their role in the cell cycle. Interestingly, PCD1 is targeted exactly four times, twice by cell cycle specific regulatory events, and twice by stress response regulatory events. This is consistent with its functional role in the cell as well as its expression levels – both synchronized with other genes in the cell cycle expression set and correlated with other stress-related genes in stress-inducing conditions.

While, as noted above, the expression levels of MBP1 and SUT1 are uncorrelated with their target genes, each is well known to play a role in its corresponding condition-specific context: the G1/S phase transition of the cell cycle for MBP1 [58], [59] and cell growth under aerobic conditions for SUT1 [60], respectively. These factors highlight PANDA’s ability to uncover regulatory events in the absence of regulator-target co-expression events. Recall that the algorithm investigates the co-expression between the *targets* of a transcription factor, rather than between the transcription factor and an individual target gene. The reason that PANDA was able to identify these factors is because their *target* genes are coexpressed. Such relationships cannot be uncovered by traditional regulator-target coexpression analysis.

Each run of PANDA predicts three networks: regulatory (*W_ij_*), co-regulatory (*C_ij_*), and cooperativity (*P_ij_*), representing, in a heuristic manner, the likelihood that two genes are co-regulated, the likelihood that an individual TF regulates a particular gene, and the likelihood that two TFs work together to cooperatively regulate their targets, respectively. As a consequence, for each expression dataset used, in addition to a regulatory network discussed above, we also have condition-specific co-regulatory and protein-cooperativity networks. We identified the top edges predicted by PANDA for each of these networks using either the cell cycle or stress response expression data, and visualized the regulatory modules surrounding the condition-specific genes identified above (Figure 4B–C). There is high connectivity within these modules, reflective of the fact that we selected genes and transcription factors based on their relatively high number of regulatory interactions. Each module contains TFs that cooperate together to regulate a common set of genes that belong to similar biological pathways. For example, SWI6 is known to complex with MBP1 [61], [62]. Interestingly, although investigation of the literature did not reveal any known direct physical interactions between MSN4, SUT1 or ADR1, there is some evidence that they might cooperate together under stress conditions. For example, both MSN4 and SUT1 are known to interact with HEK2 [63], which has been identified as a potential “bridge” protein between unstressed and heat-shocked protein-interaction networks [64].

The genes targeted in the subnetworks are also consistent with yeast biology. For example, MSH6 and UNG1 are both highly targeted only in the cell-cycle specific subnetwork and are important in mitosis and meiosis and cell cycle progression. Similarly, IKS1 and MLF3, identified in the stress-response subnetwork, are associated with functions related to heat stress and drug-response, respectively. These regulatory modules highlight PANDA’s strength in effectively integrating information from distinct data types to infer condition-specific regulatory programs and their underlying biological mechanisms.

### An Integrated Genome-wide Regulatory Network for Yeast

**Figure 5:**
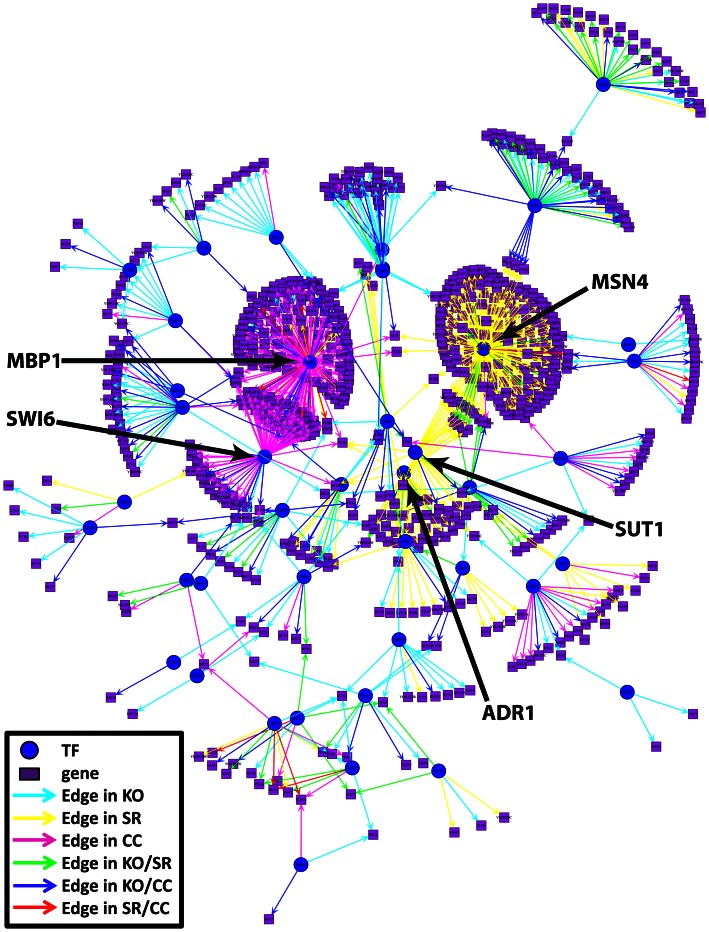
The top edges predicted by PANDA, excluding those common among all three predicted networks. Several key TFs have been identified and labeled, including the cell cycle regulator MBP1 and the stress response factor MSN4.

Finally, to gain a more complete picture of the yeast regulatory network, we integrated information from the networks predicted by PANDA in each of the three expression conditions and, excluding predictions common to all three, present the results in a single plot (Figure 5). This integrated network contains not only the regulatory edges from the highly-connected subnetworks of Figure 4B–C but also additional edges bridging these modules. Several features for the integrated networks immediately stand out in this visualization, including those already hinted at in the condition-specific modules. For example, a cluster of edges unique to the cell cycle (shown in magenta) surround MBP1, a TF that is important for the transition from G1 to S phase [58], [59]. This TF shares many of its targets with SWI6, which, as mentioned previously, is known to complex with MBP1 [61], [62]. A group of edges unique to the stress response network (shown in yellow) surround MSN4, that together with MSN2 regulates the general stress response in yeast [65]. In contrast to the stress-related and cell cycle related edges, edges predicted uniquely by the regulator knock-out dataset (shown in cyan) are spread throughout the network and can be attributed to the perturbation of a large number of functionally unrelated TFs. Interestingly, in an analogous visualization of the networks predicted by CLR (Figure S4), although the network overall is harder to discern because there are many more edges (due to the low edge overlap, see Figure 3E), it is obvious that various types of edges still tend to cluster together. This is consistent with CLR’s ability, although limited, to identify some condition-specific information (see Figure 3G and Figure S3C).

## Discussion

There has been much excitement about the potential of gene regulatory network inference methods to identify fundamental features of biological systems. An informative network model must account for the complexities of emergent biological behavior while still being simple enough to allow reasonable interpretation of the results. Here we show that message-passing provides a framework for effective integration of diverse data types. By using yeast data as a proof-of-concept, we demonstrated that the networks predicted by PANDA are not only of higher quality than those predicted by several other widely-used network reconstruction approaches, but also accurately reflect biological responses specific to each of the conditions the input expression data sets were designed to measure.

We suggest that PANDA’s ability to uncover condition-specific regulatory modules, which were not discernable using the other reconstruction algorithms, may reflect the “soft coupling” between interaction partners in the PANDA model. PANDA investigates both upstream and downstream regulatory events through simultaneous investigation of the protein cooperativity and co-regulation networks. This allows for imperfect correlation by assuming that each gene is potentially regulated by multiple TFs and explicitly including that in the underlying model. In contrast, SEREND focuses on the targets of each TF separately, rather than both the targets of a TF and the TFs targeting a gene, thus potentially missing vital information regarding complex regulatory events common in biological networks. Algorithms such as CLR generally assume that TFs and their targets are co-expressed. In contrast, PANDA instead investigates the consistency between the expression profiles of a set of genes targeted by a particular TF. ReMoDiscovery similarly looks for co-expression modules, but unlike PANDA, which updates co-expression events with co-regulation information, it requires that all genes within a module share a high similarity in their expression profiles. This can lead to potentially misclassifying genes involved in multiple biological pathways since the expression levels of these genes may not be highly correlated with the genes in any individual module but only loosely correlated with genes in several different modules.

One key aspect of PANDA is its emphasis on agreement among network neighborhoods rather than direct targeting information. For example, the algorithm infers TF-target relationships primarily from evidence that is *not* direct binding or co-expression of regulators and targets. Instead, a gene product observed to share similar interaction partners with a known target, or to be co-expressed with a targeted module, is more likely to be chosen as a new target. Not every member of a regulatory module must interact directly with every member of a downstream target, but their components can still be inferred to form a cohesive biological circuit. As the network models representing each data-type are updated, they slowly accumulate evidence from all other data sources, moving to consensus networks that represent all of the available evidence in order to explain the overall biological response. The final result is a network model that “averages” over different types of data to produce a meaningful model of the interactions those data represent. This is best reflected in PANDA’s ability to resolve networks for distinct subsets of input expression conditions. This harmonization of multiple biological data types with an underlying process occurs in tandem with the recovery of regulatory relationships under specific conditions, providing a more complete picture of biological pathway activity. This method thus serves to infer comprehensive new biology that would not be obvious based on any single data-type.

There are still a number of limitations of the PANDA method. First, the convergence of the iteration procedure requires the introduction of an “annealing” parameter α, whose value affects the configuration of the final regulatory network. Second, unlike the original message-passing paper [41], our PANDA algorithm is only heuristic and does not have an exact probabilistic interpretation. We plan to resolve these limitations in future work.

Although the analysis we present here is for yeast, the PANDA method is generally applicable to other species. For example, in preliminary studies using human data (unpublished), in a manner similar to those presented here we have successfully identified condition-specific regulatory information that accurately reflects either the particular tissue and/or disease-type in question. More importantly, the message passing algorithm at the heart of PANDA is further generalizable to different or additional data types. For example, one interesting perspective is to further integrate epigenomic profiling data that provide important enhancer activity information. In future work we will further extend PANDA to incorporate additional data-types. The major strength of PANDA is that it has provided a unified approach to make such extensions possible. We believe this overall approach, which captures the context-specific nature of communication in cell signaling networks, has tremendous potential to model biological systems and represents an important step forward in the development of integrated systems biology approaches.

## Supporting Information

Figure S1
**(A)** A plot of the AUC of the final regulatory network predicted by PANDA using the same motif and PPI data but different input sets of expression data, and across various values of the tuning parameter α. The quality of the final predicted networks is fairly similar for values of α less than approximately 0.2 but begins to rapidly decrease for the cell-cycle and stress-response networks when α is much larger than about 0.3. **(B)** A plot of the hamming distance between the network predicted at each iteration (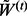) and the network at the previous iteration (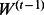), as a function of the iteration step (*t*). There is a clear transition where PANDA is “learning” a network (from approximately steps 1–40) and then where the algorithm rapidly converges (step 50 onward). This is consistent with the shapes of the learning curves shown in Figure 2A. We terminated the message-passing process once the hamming distance was less than 10^−5^.(TIF)

Figure S2 **Various assessments of the performance of PANDA compared to other reconstruction approaches. (A–B)** The AUC of the edge-weights predicted by PANDA, SEREND, ReMoDiscovery, CLR and C3Net, evaluated separately for edges that are **(A)** contained in the motif prior and **(B)** not contained in the motif prior. This analysis should mask any enhancement of AUC gained solely from the addition of motif data with the expression data and highlight the predictive power gained from the integrative message-passing approach employed by PANDA. PANDA does demonstratively better than all the other reconstruction approaches on edges that are contained in the motif prior and does comparatively or better than ReMoDiscovery, CLR and C3Net on edges that are not contained in the motif prior. SEREND has the best overall performance on these non-motif edges. **(C)** The AUC, sensitivity and precision (reported at a 90% specificity) for PANDA, SEREND and CLR when each algorithm includes data from exactly expression and motif data, excluding PPI information from PANDA and integrating motif data into the CLR predictions. **(D)** The AUC, sensitivity and precision (reported at a 90% specificity) for the networks predicted by PANDA when using either the T_Z_ (PANDA) or T_2_ (Modified) similarity scores to determine the size of the messages being passed (see Methods S1 for more description regarding these two scores). The results are very similar illustrating that PANDA is insensitive to small modifications in the similarity score used to calculate the messages being passed. **(E)** The specificity, sensitivity and precision for the top 1000 edges for the networks predicted by PANDA, SEREND, CLR and CLR+motif.
(TIF)

Figure S3 **(A)** The top 1000 edges by weight in the CLR+motif integrated network. **(B)** Functional analysis of genes belonging to each of the networks defined by all top 1000 edges identified in each of the conditional networks predicted by either PANDA, SEREND, CLR or CLR+motif. GO categories enriched at Benjamini-Hochberg FDR less than 10^−5^ and which contain at least 10% of the members in one of the condition-specific gene sets are shown. No categories were enriched at this level for genes belonging to the networks identified by SEREND. **(C)** Functional analysis of genes belonging to each of the condition-specific subnetworks identified with PANDA, SEREND, CLR or CLR+motif. Compared to Figure 3F–G, GO categories in this figure were selected if they were enriched at Benjamini-Hochberg FDR less than 10^−3^, with no percentage criteria. A few categories can now be seen enriched in the SEREND subnetworks and more categories are identified with the CLR stress subnetwork.
(TIF)

Figure S4 **The top edges predicted by CLR, excluding those common among all three predicted networks.**
(TIF)

Methods S1 **Document containing additional information regarding the implementation and evaluation of the PANDA message-passing approach.**
(PDF)

Materials S1 **Archive containing PANDA code, input data and predicted networks.**
(TGZ)
