# Supplementary figures and images for: Passing Messages between Biological Networks to Refine Predicted Interactions
Source: PLoS One. 2013 May 31;8(5):e64832. doi: 10.1371/journal.pone.0064832 (PMC3669401; doi:10.1371/journal.pone.0064832)

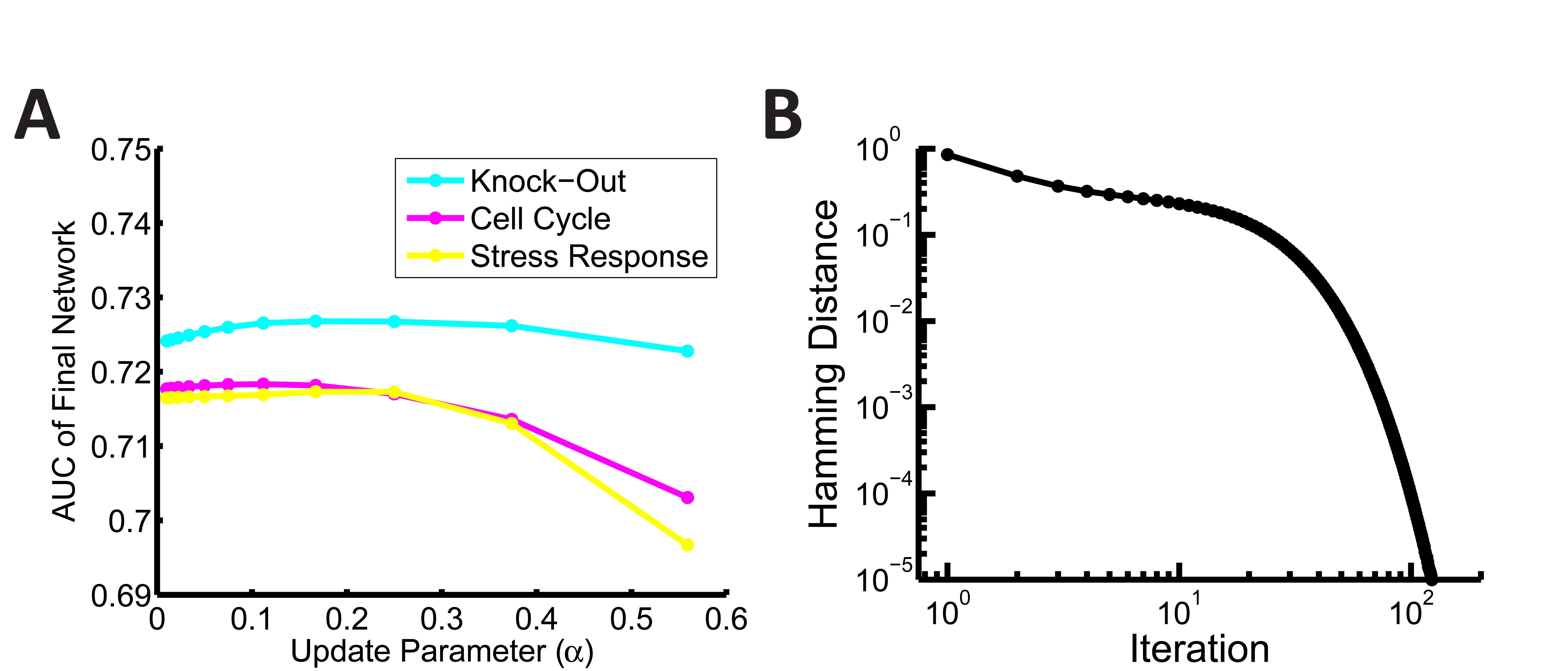

Supplement: Figure S1 — (A) A plot of the AUC of the final regulatory network predicted by PANDA using the same motif and PPI data but different input sets of expression data, and across various values of the tuning parameter α. The quality of the final predicted networks is fairly similar for values of α less than approximately 0.2 but begins to rapidly decrease for the cell-cycle and stress-response networks when α is much larger than about 0.3. (B) A plot of the hamming distance between the network predicted at each iteration () and the network at the previous iteration (), as a function of the iteration step (t). There is a clear transition where PANDA is “learning” a network (from approximately steps 1–40) and then where the algorithm rapidly converges (step 50 onward). This is consistent with the shapes of the learning curves shown in Figure 2A. We terminated the message-passing process once the hamming distance was less than 10−5. (TIF) [file pone.0064832.s001.tif]

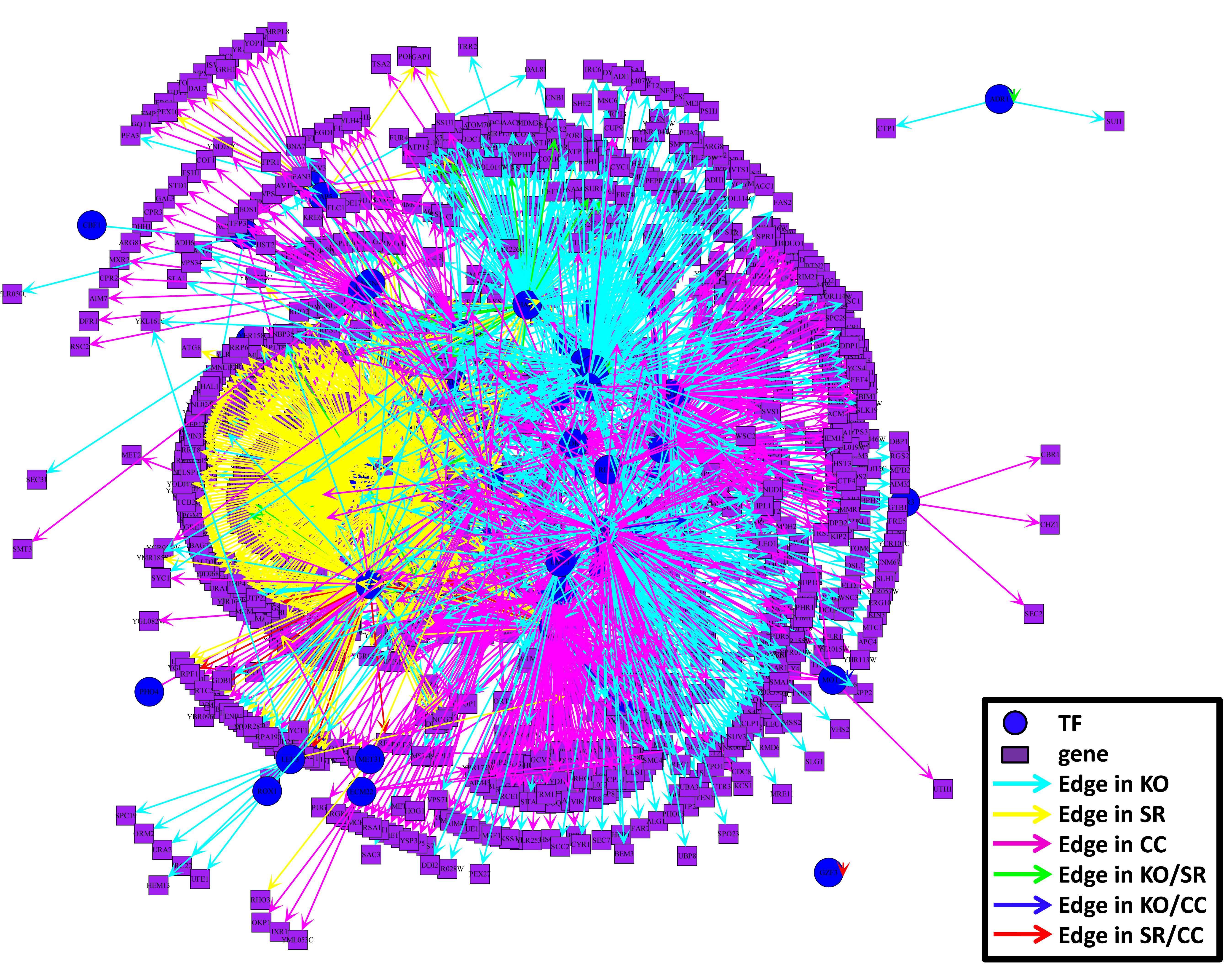

Supplement: Figure S4 — The top edges predicted by CLR, excluding those common among all three predicted networks. (TIF) [file pone.0064832.s004.tif]
