# Supplementary material for: Passing Messages between Biological Networks to Refine Predicted Interactions
Source: PLoS One. 2013 May 31;8(5):e64832. doi: 10.1371/journal.pone.0064832 (PMC3669401; doi:10.1371/journal.pone.0064832)
Supplement: Methods S1 — Document containing additional information regarding the implementation and evaluation of the PANDA message-passing approach. (PDF) [file pone.0064832.s005.pdf]

Supplemental Methods.

This supplement primarily provides elaboration on and additional motivation for the PANDA message-passing approach outlined in "**Passing messages between biological networks to refine predicted interactions**" We also include some of the details concerning the implementation of other network reconstruction algorithms as well as some additional details about the evaluation of the networks predicted by these approaches.

Data Collection/ Network Initialization

We collected data pertaining to regulatory interactions predictions or validations, including TF sequence motifs, ChIP-chip, gene expression levels, as well as protein-protein interactions for *Saccharomyces cerevisiae*, or Baker's yeast (see Table 1 from the main text as well the following for exact data-sources). We chose this as our model system as there is a large amount of data that exists for the species as well as a sufficient amount of biological literature with which to verify the biological predictions made by our model.

Motif Data (Regulatory Network)

Data: The sequence motifs and genome-wide matching sites for 204 transcription factors were obtained from the literature [1]. Neither DNA sequence conservation nor ChIP-chip data was used in identifying these motif-matching sites [2]. To remove the uncertainty of promoter-gene association, we only considered the 4,360 genes with tandem promoters in our analysis.

Initial Network Construction: This motif dataset includes 99,284 interactions of transcription factors with their predicted target genes. We included only those genes for which we had expression data (98 TFs and 2560 genes, see below). We further excluded TFs that do not have a predicted binding site in at least one of these genes as well as genes that were not predicted as targets of at least one of these TFs. This produces an initial regulatory network containing 34,128 interactions between 53 TFs and 2,555 genes predicted based only on the motif information, defined as:

$$W_{ij}^{(0)} = \begin{cases} 1 & \text{if the motif of TF } i \text{ is in the control region of gene } j \\ 0 & \text{otherwise} \end{cases} \quad (1)$$

Final Network Validation: To validate our predicted regulatory network, we downloaded ChIP-chip experiment data, that provide genome-wide information of the *in vivo* binding sites of the TFs [1]. TF targets were defined using the criterion p<0.001. We used this information to construct a "gold-standard" for our regulatory network as follows:

$$W_{ij}^{(G)} = \begin{cases} 1 & \text{if TF } i \text{ binds to the control region of gene } j \text{ with } p < 10^{-3} \\ 0 & \text{otherwise} \end{cases} \quad (2)$$

When evaluating the quality of a network against this standard we only used TFs and genes included in the standard. In other words, when calculating the AUC we excluded edges connected to TFs for which the rows of $W^{(G)}$ sum to zero and edges connected to genes for which the columns of $W^{(G)}$ sum to zero.

Gene Expression Data (Co-regulatory network)

Data: In this work we evaluate the performance of our algorithm on three representative, independently derived expression data sets, one that includes 106 experiments in which individual transcription factors have

either been knocked out or over-expressed [3], one that includes 56 experiments conducted on a synchronized cell population over a time-course [4,5], and one that includes 173 experiments where yeast cells have been exposed to many different stress-inducing conditions [6]. For each of these three data-sets, we downloaded normalized data provided by the authors on their websites [7-9].

Initial Network Construction: We began with the 2,560 genes that have expression data in all three of these data-sets and then removed genes that did not have upstream TF binding sites (see above). This resulted in a set of 2,555 genes around which we constructed our "seed" co-regulation networks. For each of the three datasets we calculated the correlation between the expression profiles of each pair of genes, i and j, using the Pearson correlation coefficient C_{kj}:

$$C_{kj}^{(0)} = \frac{\sum_l (x_{jl} - \bar{x}_{j.})(x_{kl} - \bar{x}_{k.})}{\sqrt{\sum_l (x_{jl} - \bar{x}_{j.})^2 \sum_l (x_{kl} - \bar{x}_{k.})^2}}. \tag{3}$$

where x_{jl} is the expression level of gene j in condition l and $\bar{x}_{j.}$ is the average expression level of gene j across all conditions. This created three initial co-regulatory networks, one for each expression dataset. Note that a gene is always co-expressed with itself, therefore $C_{jj}=1$. In the rare absence of expression data, this assignment can be used to initialize C to an identity matrix. It also allows us to account for genes in our model that are the only target of a TF and thus are only co-regulated with themselves and not other genes.

Final Network Validation: We used experimentally-derived binding sites from ChIP-chip [1] to construct a "gold-standard" to evaluate our co-regulatory network. For every gene-pair, we determined if those two genes were ever targeted by the same transcription factor in the ChIP data and defined the network as follows:

$$C_{kj}^{(G)} = \begin{cases} 1 & \text{if any TF binds to the control regions of both genes } j \text{ and } k \text{ with } p < 10^{-3} \\ 0 & \text{otherwise} \end{cases} \tag{4}$$

When evaluating the quality of a network against this gold standard we only used genes with information in the standard. In other words, when calculating the AUC we excluded edges connected to genes for which the rows of $C^{(G)}$ sum to zero.

Protein-Protein Interaction Data (Protein-Cooperativity Network)

Data: We downloaded protein-protein interactions from NCBI [10]. This repository includes interactions reported in the BioGrid database [11,12].

Initial Network Construction: We filtered the data set to include only physical interactions between the 53 TFs in the motif network (see above) with evidence from high-throughput Affinity Capture-MS experiments, and defined the initial network as:

$$P_{im}^{(0)} = \begin{cases} 1 & \text{if there is TAP-MS evidence that TF } i \text{ interacts with TF } m \\ 0 & \text{otherwise} \end{cases}. \tag{5}$$

We also set $P_{ii}=1$, since a TF can be viewed as co-operating with itself. In the absence of protein-protein interaction data, P is initialized to the identity matrix.

Final Network Validation: Separately, we filtered the protein-protein interactions listed in the above database to include only physical interactions between the 53 TFs in the motif network with evidence codes from "low-throughput" (and more stringent) experiments, including "co-fractionation," "co-localization," "FRET," and "reconstituted complex." These interactions define our "gold-standard":

$$P_{im}^{(G)} = \begin{cases} 1 & \text{if there is low-throughput evidence that TF } i \text{ interacts with TF } m \\ 0 & \text{otherwise} \end{cases} \quad (6)$$

As with the regulatory and co-regulatory networks, the quality of the final networks was only evaluated using edges connected to TFs for which we had information in our gold-standard, thereby excluding from the evaluation any edges connected to TFs for which the rows of $P^{(G)}$ sum to zero.

Initial Network Normalization

In order to pass information between data types that are intrinsically different, $C^{(0)}$, $W^{(0)}$ and $P^{(0)}$ are all normalized by converting to Z-scores, as follows:

$$X_{pq}^{(Z)} = Z(X_{pq}) = \frac{1}{\sqrt{2}} Z_p(X_{pq}^{(0)}) + \frac{1}{\sqrt{2}} Z_q(X_{pq}^{(0)}), \quad (7)$$

where $X_{pq}^{(0)}$ represent the entry in the pth row and q^{th} column of data type X. For example if $X=W$ then, for an edge W_{ij} in W:

$$W_{ij}^{(Z)} = Z(W_{ij}^{(0)}) = \frac{1}{\sqrt{2}} Z_i(W_{ij}^{(0)}) + \frac{1}{\sqrt{2}} Z_j(W_{ij}^{(0)}), \quad (8)$$

and furthermore,

$$Z_i(W_{ij}^{(0)}) = \frac{W_{ij}^{(0)} - \mu_i}{\sigma_i}, \quad (9)$$

where μ_i and σ_i represent the mean and standard deviation across row i in W. This transformation is integrated into the PANDA algorithm and occurs only once for each data type — immediately after network initialization and before the first message is passed.

Finding Agreement Between Two Networks

Before discussing the specifics of the PANDA algorithm, we wish to offer some conceptual motivation for the mathematical approach. PANDA aims to find *agreement* between the data represented by two networks. In other words, we give more weight to an edge if there is a large amount of agreement between data representing that edge, and subtract weight from an edge if there is a large amount of disagreement between data representing that edge.

With this in mind, we use a similarity metric in order to evaluate the potential weight of an edge in each of our networks. We remind the reader that in order to put the networks representing each of our datasets on the same scale, we normalized each by recasting edge weights into z-score units (see Equations 7-9). As a result, we need our metric to take two vectors representing z-scored edge weights and return a score whose value is also in z-score units, or at least whose value can be interpreted in much the same way as the values of the input vectors.

We base the similarity score used by PANDA on the Tanimoto similarity:

$$T(\vec{x}, \vec{y}) = \frac{\vec{x} \cdot \vec{y}}{\|\vec{x}\|^2 + \|\vec{y}\|^2 - \vec{x} \cdot \vec{y}} = \frac{\sum_i x_i y_i}{\sum_i x_i^2 + \sum_i y_i^2 - \sum_i x_i y_i}. \quad (10)$$

This metric will return a value of one for perfect agreement and a value close to zero for no agreement. We modify the above equation in two simple ways such that, given $\vec{x}$ and $\vec{y}$ in z-score units, the similarity value returned will also mimic z-score units. First, we force the distribution to be symmetric around 0 (in other words, for $\vec{y}' = -\vec{y}, T_Z(\vec{x},\vec{y}') = -T_Z(\vec{x},\vec{y})$) by the addition of absolute value signs around the final dot product in the denominator. Then we add a square root around the entire denominator such that the returned values will no longer be strictly bounded between [-1,1] but may take any real values. Namely:

$$T_Z(\vec{x},\vec{y}) = \frac{\vec{x}\cdot\vec{y}}{\sqrt{\|\vec{x}\|^2 + \|\vec{y}\|^2 - |\vec{x}\cdot\vec{y}|}} = \frac{\sum_i x_i y_i}{\sqrt{\sum_i x_i^2 + \sum_i y_i^2 - \left|\sum_i x_i y_i\right|}} . \quad (11)$$

One can immediately observe that, given $\vec{x}$ and $\vec{y}$ in units of z-score, the numerator is in units of z-scored-squared and the denominator is in units of z-score, thus T_Z will be in units of z-score (instead of unit-less as in Equation 10). We also note that the maximum and minimum values that can be obtained when z-scoring a vector of values are equal to $\sqrt{N-1}$ and $-\sqrt{N-1}$, respectively, and occur when all entries of a vector except one have the same value. We would like T_Z to return these maximum and minimum values when x and y either perfectly agree or perfectly disagree. We point out that, because variance of a vector in units of z-score is equal to one:

$$\left\langle x^2 \right\rangle = 1, \text{ and } \sum_i x_i^2 = N . \quad (12)$$

Using this identity, we observe that for $\vec{x} = \vec{y}$, T_Z equals $\sqrt{N}$ and for $\vec{x} = -\vec{y}$, $T_Z = -\sqrt{N}$, which approximates these maximum and minimum values. Of course, because we simultaneously z-score each row and column of the initial networks (see Equations 7-9), the values are only approximately in z-score units; however, we believe that the above gives an intuitive way to interpret the similarity measure values.

As the contribution of the denominator is mostly to keep the values of T_Z in the same range of values as z-scores, one might desire to simplify the mathematical form of this equation, for example, by introducing a factor of 2 in front of the last element of the denominator (compare Equation 11 to Equation 13). Although prettier to write, this additional factor mitigates some of the properties of T_Z described above. For example, instead of being bounded, there is now a singularity at $\vec{x} = \vec{y}$. In principle this is not an issue, but in practice, especially as we *expect* to find agreement between the networks derived from real data, and furthermore, the message-passing procedure described below actually updates each network to make it more in agreement with the others, this additional factor of two also necessitates the addition of a small value, ε, to the equation to avoid computational anomalies:

$$T_2(\vec{x},\vec{y}) = \frac{\vec{x}\cdot\vec{y}}{\sqrt{\|\vec{x}\|^2 + \|\vec{y}\|^2 - 2|\vec{x}\cdot\vec{y}|} + \varepsilon} = \frac{\vec{x}\cdot\vec{y}}{\min(\|\vec{x}+\vec{y}\|, \|\vec{x}-\vec{y}\|) + \varepsilon} . \quad (13)$$

We have run PANDA (see below) using both T_Z and T_2 (using ε =1e-10) and observe little difference between the results (see Supplemental Figure 2D). Therefore, to avoid the potential singularity, we prefer using T_Z (Equation 11) but inform the reader that either T_Z or T_2 will produce networks that are highly informative of a given biological system.

PANDA: Passing Attributes between Networks for Data Assimilation

After constructing initial networks for each data type, we pass messages between these networks in order to inform and update the values of their edges to represent more functionally relevant predictions. The message-passing occurs in two steps: (1) estimate and update the regulatory network, and (2) estimate and update the data-specific (co-regulation and protein-cooperativity) networks. These updates are iteratively repeated until convergence.

Estimate and Update the Regulatory Network

Using Protein Interactions to Predict the Responsibility of Regulatory Relationships: When regulating a gene, transcription factor proteins rarely act alone. Instead, they often interact to form complexes that, in turn can bind to the control regions of genes and recruit transcriptional units such as RNA polymerase at a level unattainable by any individual portion of the complex. The structure of complexes allows transcription factors to influence the expression level of a gene in the absence of a physical binding site in the control region of that gene.

We assume our protein-protein interaction network represents cooperative regulation by TFs wherein the strength of an edge between two TFs is indicative of how often those two transcription factors work together to regulate the expression levels of genes. With this in mind, we heuristically combine the regulatory network (W) with the protein-cooperativity network (P) to predict the *responsibility* ($R_{ij}^{(t)}$) of an edge from TF i to gene j in the regulatory network. Namely, since TFs that cooperatively regulate their targets share responsibility for the behavior of these genes, to determine the responsibility an individual TF has in regulating a particular target gene, we determine the level of agreement between the TFs that target gene j ($W_{.j}^{(t)}$), and those that form a complex with TF i ($P_{i.}^{(t)}$):

$$R_{ij}^{(t)} = T_Z(P_{i.}^{(t)}, W_{.j}^{(t)}) = \frac{\sum_m P_{im}^{(t)} W_{mj}^{(t)}}{\sqrt{\sum_m \left(P_{im}^{(t)}\right)^2 + \sum_m \left(W_{mj}^{(t)}\right)^2 - \left|\sum_m P_{im}^{(t)} W_{mj}^{(t)}\right|}} . \tag{14}$$

This formula was motivated by our desire to approximately maintain the resulting values to have the standard normal distribution. Intuitively, this is achieved by having the numerator in units of Z-scored-squared (rows and columns of P and W are approximately standardized, see Equations 7-9) and the denominator in units of Z-score. For example, if row i of P and column j of W are equal, then R_{ij} will be equal to the magnitude of this row/column in P/W. The same intuitive approach is used for other terms, including the *availability* (Equation 15), the co-regulatory network (Equation 17) and the protein-cooperativity network (Equation 18).

Using Co-regulation to Predict the Availability of Regulatory Relationships: Correlation in the expression profiles of two genes can be indicative of many types of relationships between those genes, including both direct (i.e. regulation of a gene by a transcription factor) and indirect relationships. We treat expression correlation as a “co-regulation” network, where values indicate the degree to which two genes are targeted by the same set of transcription factors. Under this assumption, we combine information in the regulatory network (W) with the co-regulatory network (C) to predict the *availability* (A_{ij}) of an edge between TF i and gene j in the regulatory network. Namely, since genes that are targeted by the same TF are co-regulated, at each iteration, t, to calculate $A_{ij}^{(t)}$ we determine the level of agreement between the regulatory targets of TF i ($Wi_{.}^{(t)}$) and the set of genes with which gene j is co-regulated ($C_{.j}^{(t)}$):

$$A_{ij}^{(t)} = T_Z(W_{i.}^{(t)}, C_{.j}^{(t)}) = \frac{\sum_k W_{ik}^{(t)} C_{kj}^{(t)}}{\sqrt{\sum_k \left(W_{ik}^{(t)}\right)^2 + \sum_k \left(C_{kj}^{(t)}\right)^2 - \left|\sum_k W_{ik}^{(t)} C_{kj}^{(t)}\right|}} . \quad (15)$$

Combining the Availability and Responsibility and Updating the Regulatory Network: Since regulation requires both that a TF is responsible for the regulatory status of its target gene and that the target gene is available to be regulated by that TF, we use the average of these two values ($\tilde{W}_{ij}^{(t)} = 0.5A_{ij}^{(t)} + 0.5R_{ij}^{(t)}$) and update the regulatory network by a small amount (α; $0<\alpha<1$):

$$W_{ij}^{(t+1)} = (1-\alpha)W_{ij}^{(t)} + \alpha\tilde{W}_{ij}^{(t)} , \quad (16)$$

where the above equation represents the weighted average between the previous estimate for the regulatory network and the current estimate. The update parameter, α, can therefore be used to tune how quickly messages are being passed. We have found that in practice results are fairly stable for $0<\alpha<0.2$ (see Supplemental Figure 2B). In order to determine converge, at each iteration step we also calculate the hamming distance between the current and estimated network:

$$H^{(t)} = \left|\tilde{W}^{(t)} - W^{(t-1)}\right| = \frac{1}{N}\sum_{i,j}\left|\tilde{W}_{ij}^{(t)} - W_{ij}^{(t-1)}\right| , \quad (16)$$

Where N is the number of possible edges in the regulatory network, or the product of the number of genes times the number of TFs considered by the algorithm. In this work we define convergence when $H^{(t)}$ is less than 10^{-5}.

Incorporating Further Data-types Into the Regulatory Network Estimate: The above process can be expanded or modified to incorporate additional or different data-types that enhance our ability to estimate either a TF's responsibility in regulating a gene, or a gene's availability to be regulated by a TF. Additional data-types could be included by estimating additional values for either the availability or responsibility of the edges and merging these values together when updating W. Data-types besides the ones outlined here may also be used to estimate the availability and responsibility of the edges in the regulatory network. In these cases the exact formulation of equations above may need to change to better represent the information in these data types.

Estimate and Update the Co-regulatory and Protein-cooperativity Networks

We not only pass messages between TFs and their targets, but also incorporate information from the regulatory network into the information represented in the co-regulation and protein-cooperativity networks.

Using Regulatory Relationships to Predict Co-regulation: The co-regulation network is initialized based on gene expression correlation. We admit, however, that the assumption that co-expression is equivalent to co-regulation is a simplification. Since co-regulated genes are, by definition, targeted by the same TFs, we can improve the quality of our co-regulation network and estimate the weight of an edge between two genes, j and k, in the co-regulation network (C_{jk}) by comparing the set of TFs targeting gene j ($W_{.j}$) with the set of TFs targeting gene k ($W_{.k}$):

$$\tilde{C}_{kj}^{(t)} = T_Z(W_{.k}^{(t)}, W_{.j}^{(t)}) = \frac{\sum_i W_{ik}^{(t)} W_{ij}^{(t)}}{\sqrt{\sum_i \left(W_{ik}^{(t)}\right)^2 + \sum_i \left(W_{ij}^{(t)}\right)^2 - \left|\sum_i W_{ik}^{(t)} W_{ij}^{(t)}\right|}} . \quad (17)$$

Since a gene is, by definition, always co-regulated with itself, we use the self-co-regulation of a gene j (C_{jj}) to represent the amount of certainty we have in the edges surrounding that gene. $\tilde{C}_{jj}^{(t)} = \sigma_j N_G e^{2\alpha t}$ where N_G is the number of genes queried by PANDA and σ_j is the standard deviation across $C_{.j}$. In principle, since we are dealing with Z-scores, σ_j should be equal to one, however in practice we have found that it can vary enough to interfere with the message-passing. The value of $\tilde{C}_{jj}^{(t)}$ increases as messages are being passed such that eventually the self-regulation will dominate the availability equation (Equation 15), $A_{ij}^{(t)} = W_{ij}^{(t)}$, and the algorithm will converge around some estimate of edges.

Using Regulatory Relationships to Predict TF Interactions: While a large number of TF interactions have been identified in various experimental assays, only a small fraction occur *in vivo* and are functionally relevant, while still other TFs may not directly physically interact, but still may need to both be present for the activation of their target genes. Since TFs that target the same sets of genes are likely to co-operate together to regulate those genes, we can estimate the weight of an edge between two TFs, i and m, in the protein-cooperativity network (P_{im}) by comparing the set of genes regulated by TF i to those regulated by TF m:

$$\tilde{P}_{im}^{(t)} = T_Z(W_{i.}^{(t)}, W_{m.}^{(t)}) = \frac{\sum_j W_{ij}^{(t)} W_{mj}^{(t)}}{\sqrt{\sum_j \left(W_{ij}^{(t)}\right)^2 + \sum_j \left(W_{mj}^{(t)}\right)^2 - \left|\sum_j W_{ij}^{(t)} W_{mj}^{(t)}\right|}} . \quad (18)$$

Since a TF, by definition, always regulates the same set of genes as itself, we use the self-cooperativity of a TF (P_{ii}) to represent the amount of certainty we have in the edges surrounding that TF. $\tilde{P}_{ii}^{(t)} = \sigma_i N_T e^{2\alpha t}$ where N_T is the number of TFs queried by PANDA and σ_i is the standard deviation across $P_{i.}$. In principle, since we are dealing with Z-scores, σ_i should be equal to one, however in practice we have found, especially for smaller samples of TFs, that it can vary enough to interfere with the message-passing. The value of $\tilde{P}_{ii}^{(t)}$ increases as messages are being passed such that eventually self-cooperation will dominate the responsibility equation (Equation 14), $R_{ij}^{(t)} = W_{ij}^{(t)}$, and the algorithm will converge around some estimate of edges.

Network Updates: This process gives estimates for the co-regulation and protein-cooperativity networks that are in agreement with what is known about the regulatory network. We use these values to update C and P:

$$C_{kj}^{(t+1)} = (1-\alpha) C_{kj}^{(t)} + \alpha \tilde{C}_{kj}^{(t)} \quad (19)$$

$$P_{im}^{(t+1)} = (1-\alpha) P_{im}^{(t)} + \alpha \tilde{P}_{im}^{(t)} . \quad (20)$$

The same value for α as was used to pass-messages to the regulatory network is also used to pass messages to these networks.

Incorporating Further Data types: In this work we only explored how regulatory information might be reflected in co-regulation (derived from co-expression) and protein interactions, however, other data-types will reflect regulatory information as well, in which case additional data types could be added to the PANDA model and updated along with the co-regulation and protein-cooperativity networks.

Network Validation

We evaluated the quality of the predicted networks using AUC-ROC (AUC for short) statistics. When running PANDA, the initial "seed" networks (Equations 1, 3 and 5 above) already have a quality greater than random. Therefore we evaluated the significance of the improvement in the AUC of the final predicted networks compared to the initial "seed" networks using a jackknife procedure in which we removed motif, interaction and expression data regarding a random 10% of TFs and 10% of genes and ran PANDA on the remaining data. We repeated this 100 times in order to generate a distribution of initial and final AUC scores for each network. We determined the significance of the difference in these two distributions by taking the pair-wise difference between the AUCs of the final and initial networks. We assumed that these differences should follow a normal distribution, and likewise determined their mean and standard deviation. The significance of the difference between these values and that of no improvement (a difference value of zero) was calculated by taking the ratio of the mean over the standard deviation and determining the CDF of this value compared to a normal distribution with a mean of zero and standard deviation of one.

Identifying Condition-Specific Regulatory Information and modules:

We took the union of the top 1000 edges predicted by PANDA in each of the condition-specific regulatory networks to form an integrated genome-wide regulatory network for yeast (results of the analysis are similar if we take other edge cutoffs). We defined condition-specific subnetworks as the edges that appear uniquely in only one of these three edge sets. To analyze the functional properties of these condition-specific subnetworks, for each subnetwork, we took all genes or transcription factor connected any edge in the subnetwork. We performed functional analysis using the DAVID bioinformatics tool on these genes, using the 2,555 genes from our seed networks as a background.

Next, to determine specific gene-level condition-specific information within this unified network, we identified TFs and genes for which at least one-half of their predicted regulatory interactions in the unified network are identified in only one of the condition-specific subnetworks. Among these TFs and genes we selected those that have an overall connectivity of at least 10 or 3, respectively.

We wanted to visualize the regulatory information surrounding these genes and TFs. For each expression dataset, PANDA produces three predicted networks: co-regulatory, regulatory, and cooperativity. We identified condition-specific edges from the co-regulatory and cooperativity networks by thresholding based on the final edge-weight value of P and C and selecting the top 10% of edges. For consistency, we used the 1000 edges selected in the above analysis to represent the regulatory network. Finally, we build regulatory "modules" for each specific expression condition by taking the subset of these selected edges that extend between the condition-specific genes identified above. We then visualized these genes and edges to illustrate how PANDA uncovers regulatory information that can be very specific to the expression conditions used in building the model.

Running and Characterizing the Results of Other Network Reconstruction Algorithms

In addition to PANDA we ran and characterized the results of three other widely-cited network reconstruction algorithms: SEREND [13], ReMoDiscovery [14], CLR [15] and C3Net [16]. All four were implemented using default parameter settings.

CLR: The CLR algorithm[15] was downloaded from the authors' website [17] and run using default parameters. We used the same gene expression data that we fed into the PANDA algorithm (a 2,555×N_C matrix where N_C is

the number of conditions in the expression data-set being used). The output of CLR is a symmetric 2,555×2,555 gene-gene network that we reduce to a 53×2,555 TF-gene network to more correctly represent transcriptional regulation. This “reduced” network was evaluated using AUCstatistic and the same ChIP-chip standard ($W^{(G)}$, Equation 2 above) as the one used to evaluate the regulatory network predicted by PANDA.

CLR+motif: To integrate CLR with motif data, we took the scores predicted for each edge in the CLR network (see above). We then determined which of those edges do or do not exist in the motif prior (Equation 1). For edges that exist in the motif prior, we added a value to the original CLR score, equal to the maximum score obtained by an edge not in the motif prior. The result was a modified CLR-score for edges that also exist in the motif prior that is guaranteed to be greater than or equal to any of the scores for edges not are not in the motif prior. Therefore, when ordering edges based on their score in this CLR+motif network, edges that exist in the motif prior ($W_{ij}^{(0)}=1$, see Equation 1) will always be more highly ranked than those not in the motif prior ($W_{ij}^{(0)}=0$), however, within these two classes of edges (those that do exist in the motif prior, and those that do not), edges will be ordered according to their original CLR score.

C3Net: The C3Net algorithm [16] was downloaded from CRAN [18] and run in R. To estimate the regulatory network we provided the ‘c3net’ function with the same expression dataset as was provided to PANDA (a 2,555×N_C matrix where N_C is the number of conditions in the expression data-set being used). As with CLR, the model was run using the default parameter setting. The output is a 2,555×2,555 gene-gene network that we reduced to a 53×2,555 TF-gene network. This “reduced” network was evaluated using ROC statistics and the same ChIP-chip standard ($W^{(G)}$, Equation 2 above) as the one used to evaluate the regulatory network predicted by PANDA. It is worth noting that the 2,555×2,555 network predicted by C3Net is quite sparse and is made even more so by the reduction into TF-gene space, which may contribute to its relatively poor performance in the ROC evaluation.

ReMoDiscovery: The ReMoDiscovery [14] java application was downloaded from the authors’ website [19]. We provided ReMoDiscovery with the same expression data set as was provided to the other algorithms. In contrast to CLR and C3Net, ReMoDiscovery also requires a user to specify motif and ChIP-chip data. Because we wished to use ChIP-chip data as a verification set, we initialized the motif and ChIP-chip data requested by ReMoDiscovery to the same motif information we used to initialize the regulatory network ($W^{(0)}$) in the PANDA algorithm. The output of ReMoDiscovery is a file with regulatory “modules.” Each “module” includes a list of genes as well as TFs that regulate the module. We converted this output into a network by placing edges from each of the TFs identified with a module to all of the genes in that module. We evaluated this network using ROC statistics and the same ChIP-chip standard ($W^{(G)}$, Equation 2 above) as the one used to evaluate the regulatory network predicted by PANDA. It is worth noting that many of the “modules” predicted by ReMoDiscovery only contained a single gene and thus merging the results in this manner may have obscured any biological signal found by the few modules that contained multiple genes.

SEREND: The SEREND [13] java application was downloaded from the supporting website [20]. We provided the application with the same expression data set that was provided to PANDA, CLR, ReMoDiscovery and C3Net, and the same motif data that was provided to PANDA and ReMoDiscovery. The output contains several scores for every possible TF-gene pair. In the evaluation we used the score that gives the highest overall AUC, the “Meta Classifier”.

1. Harbison CT, Gordon DB, Lee TI, Rinaldi NJ, Macisaac KD, et al. (2004) Transcriptional regulatory code of a eukaryotic genome. Nature 431: 99-104.
2. http://fraenkel.mit.edu/Harbison/release_v24/txtfiles/.
3. Chua G, Morris QD, Sopko R, Robinson MD, Ryan O, et al. (2006) Identifying transcription factor functions and targets by phenotypic activation. Proc Natl Acad Sci U S A 103: 12045-12050.

4. Pramila T, Miles S, GuhaThakurta D, Jemiolo D, Breeden LL (2002) Conserved homeodomain proteins interact with MADS box protein Mcm1 to restrict ECB-dependent transcription to the M/G1 phase of the cell cycle. Genes Dev 16: 3034-3045.
5. Pramila T, Wu W, Miles S, Noble WS, Breeden LL (2006) The Forkhead transcription factor Hcm1 regulates chromosome segregation genes and fills the S-phase gap in the transcriptional circuitry of the cell cycle. Genes Dev 20: 2266-2278.
6. Gasch AP, Spellman PT, Kao CM, Carmel-Harel O, Eisen MB, et al. (2000) Genomic expression programs in the response of yeast cells to environmental changes. Mol Biol Cell 11: 4241-4257.
7. http://genome-www.stanford.edu/yeast_stress/.
8. http://hugheslab.ccbr.utoronto.ca/supplementary-data/yeastTF/.
9. http://labs.fhcrc.org/breeden/cellcycle/index.html.
10. ftp://ftp.ncbi.nlm.nih.gov/gene/GeneRIF/ (taxID#559292).
11. Stark C, Breitkreutz BJ, Chatr-Aryamontri A, Boucher L, Oughtred R, et al. (2011) The BioGRID Interaction Database: 2011 update. Nucleic Acids Res 39: D698-704.
12. Stark C, Breitkreutz BJ, Reguly T, Boucher L, Breitkreutz A, et al. (2006) BioGRID: a general repository for interaction datasets. Nucleic Acids Res 34: D535-539.
13. Ernst J, Beg QK, Kay KA, Balazsi G, Oltvai ZN, et al. (2008) A semi-supervised method for predicting transcription factor-gene interactions in Escherichia coli. PLoS Comput Biol 4: e1000044.
14. Lemmens K, Dhollander T, De Bie T, Monsieurs P, Engelen K, et al. (2006) Inferring transcriptional modules from ChIP-chip, motif and microarray data. Genome Biol 7: R37.
15. Faith JJ, Hayete B, Thaden JT, Mogno I, Wierzbowski J, et al. (2007) Large-scale mapping and validation of Escherichia coli transcriptional regulation from a compendium of expression profiles. PLoS Biol 5: e8.
16. Altay G, Emmert-Streib F (2011) Structural influence of gene networks on their inference: analysis of C3NET. Biol Direct 6: 31.
17. http://gardnerlab.bu.edu/software&tools.html.
18. http://cran.r-project.org/web/packages/c3net/index.html.
19. http://homes.esat.kuleuven.be/~kmarchal/Supplementary_Information_Lemmens_2006/Index.html.
20. http://www.cs.cmu.edu/~jernst/Ecoli/.
